# Bias and imprecision in posture percentile variables estimated from short exposure samples

**DOI:** 10.1186/1471-2288-12-36

**Published:** 2012-03-25

**Authors:** Svend Erik Mathiassen, Jens Wahlström, Mikael Forsman

**Affiliations:** 1 Centre for Musculoskeletal Research, Department of Occupational and Public Health Sciences, University of Gävle, Gävle, Sweden; 2 Department of Public Health & Clinical Medicine, Occupational and Environmental Medicine, Umeå University, Umeå, Sweden; 3 Division of Occupational and Environmental Medicine, Department of Public Health Sciences, Karolinska Institutet, Stockholm, Sweden

## Abstract

**Background:**

Upper arm postures are believed to be an important risk determinant for musculoskeletal disorder development in the neck and shoulders. The 10^th ^and 90^th ^percentiles of the angular elevation distribution have been reported in many studies as measures of neutral and extreme postural exposures, and variation has been quantified by the 10^th^-90^th ^percentile range. Further, the 50^th ^percentile is commonly reported as a measure of "average" exposure. These four variables have been estimated using samples of observed or directly measured postures, typically using sampling durations between 5 and 120 min.

**Methods:**

The present study examined the statistical properties of estimated full-shift values of the 10^th^, 50^th ^and 90^th ^percentile and the 10^th^-90^th ^percentile range of right upper arm elevation obtained from samples of seven different durations, ranging from 5 to 240 min. The sampling strategies were realized by simulation, using a parent data set of 73 full-shift, continuous inclinometer recordings among hairdressers. For each shift, sampling duration and exposure variable, the mean, standard deviation and sample dispersion limits (2.5% and 97.5%) of all possible sample estimates obtained at one minute intervals were calculated and compared to the true full-shift exposure value.

**Results:**

Estimates of the 10^th ^percentile proved to be upward biased with limited sampling, and those of the 90^th ^percentile and the percentile range, downward biased. The 50^th ^percentile was also slightly upwards biased. For all variables, bias was more severe with shorter sampling durations, and it correlated significantly with the true full-shift value for the 10^th ^and 90^th ^percentiles and the percentile range. As expected, shorter samples led to decreased precision of the estimate; sample standard deviations correlated strongly with true full-shift exposure values.

**Conclusions:**

The documented risk of pronounced bias and low precision of percentile estimates obtained from short posture samples presents a concern in ergonomics research and practice, and suggests that alternative, unbiased exposure variables should be considered if data collection resources are restricted.

## Background

### Upper arm postures and musculoskeletal disorder risk

In ergonomics intervention and epidemiology studies, upper arm posture is often assessed and viewed as an important measure of biomechanical exposure [1-4]. This is justified by evidence that working with elevated arms is associated with increased risk of musculoskeletal disorders (MSD) in the shoulders [5,6]. The extent of 'rest' during occupational work has also been proposed to be an important determinant of risk [7,8], which suggests that the occurrence of neutral postures is of interest as well [9-12]. In occupational studies, percentiles of the cumulative posture distribution have been commonly used to assess the occurrence of neutral and extreme postures. Most studies have employed the 10^th ^and 90^th ^percentiles for this purpose [12-27]. The prominent standing of these variables stems from two seminal papers, published approximately 30 years ago, in which Jonsson suggested that an exposure distribution should be described not only by its median, but also by the 10^th ^and 90^th ^percentiles to reflect its 'static' and 'peak' properties, respectively [28,29]. In these papers, Jonsson also proposed guidelines for acceptable 10^th^, 50^th ^and 90^th ^percentiles of muscle activity during occupational work. While originally proposed as a data reduction method for electromyographic recordings from relevant muscles, and extensively used for this purpose in the ergonomics literature (examples in [30]), posture percentiles *ad modum *Jonsson were introduced by Aarås [31], and have - as shown by the references above - been commonly accepted as descriptive metrics in occupational studies of biomechanical exposures. Percentiles have even been used to describe the distribution of other occupational and environmental exposures, for instance noise [32].

In addition to the effects of neutral and extreme postures in their own right, lack of posture variation is also commonly accepted to imply an increased risk for disorders, especially in the shoulders and neck [33,34]. The concept of 'variation' has been proposed to include several aspects of change in exposure over time [35], one of which - the amplitude of exposure changes - can be assessed using a percentile range for postural distribution. In studies of the upper arm, the 10^th ^-90^th ^[9,12,36,37] and 5^th^-95^th ^[13,22,38] percentile ranges have been used for this purpose.

### Posture sampling strategy, precision and bias

Occupational posture assessments by observation and direct technical recording have typically employed a continuous sampling approach of a duration ranging from some minutes up to approximately two hours [13,14,16,17,25,26,31,39]. In a few rare cases, a whole shift has been monitored [12,19,27,40,41], or even a whole day including both work and leisure [37]. These sampling strategies have been applied even in studies pursuing exposures over an extended period of time, for example, a month or a year. Since postures vary across time within individuals, limited duration samples do, in these cases, return uncertain estimates. The variability of posture variables between individuals, and within and between days within individuals has been addressed in several occupational settings [12,23,40,42,43], and the influence of sample size [30,40,44-51], sample allocation [46,48,50,52], and data processing method [53,54] on the precision of the resulting exposure estimate has been discussed for postures and other biomechanical exposures. All of these studies have addressed the size and effects of random error, assuming that the investigated sampling strategies produce unbiased exposure estimates, i.e. values free of systematic error. However, the latter conjecture is not trivial, particularly for variables that are not necessarily well estimated over an extended period of time by averaging across embedded sub-periods, for example, variances [55] and, as we propose, percentiles. Data on lumbar muscle activity in different occupations reported by Trask et al. [56] indicate that job exposure percentiles may, indeed, be biased if based on short samples. Bias and precision when assessing posture percentiles have not to our knowledge, been previously investigated, nor have the effects of the selected sampling strategy on the size and structure of these errors been quantified.

The purpose of this study was to examine the effect of different data sampling strategies, ranging in duration from 5 to 240 min, on the bias and precision of estimated full-shift values for the 10^th^, 50^th ^and 90^th ^upper arm elevation percentiles, and the 10^th^-90^th ^percentile range. We based the study on 73 full-shift inclinometry recordings from 20 hairdressers.

## Methods

### Subjects and posture recordings

Full-shift, right upper arm elevation angles were collected from a convenience sample of 20 female hairdressers in 13 salons in Umeå, Sweden; mean age 31 (range 19-60) years, mean height 167 (150-176) cm, mean seniority 11 (0.25-42) years. Only hairdressers working a minimum of 30 h per week were considered for inclusion. These data were collected as part of a larger study which included 28 hairdressers [12], however, only data from hairdressers who fully completed a study diary were included in the parent data set for the present data analyses. Participants were recorded over four full shifts within the same week, using triaxial accelerometers [57] mounted above the insertion of the right deltoid muscle, aligned with the long axis of the humerus. These accelerometers have previously been shown to provide elevation angles deviating, on average, less than 2° from true values [57]. The study was approved by the local ethical committee at Umeå University.

Procedures for calibration and basic data processing have previously been described in detail [12]. Following the exclusion of shifts with technical shortcomings and/or less than 300 min of viable data, 73 acceptable shifts remained: 14 subjects with 4 shifts, 5 subjects with 3 shifts, and 1 subject with 2 shifts. The mean full-shift recording duration was 486 min (range 300 to 595 min).

### Data processing and simulated sampling strategies

For all 73 shifts, the true 10^th^, 50^th ^and 90^th ^percentiles, and the 10^th^-90^th ^percentile range were determined from the cumulative distribution of the full-shift parent data set recording. The same four variables were also estimated for seven simulated sampling strategies of durations: 5, 10, 20, 40, 60, 120 and 240 min. For each sampling strategy, sampling windows of the appropriate duration were formed from the parent data set at one-minute intervals across the full-shift data. For example, employing the 10-minute sampling strategy over a 480 min shift, 471 ten-minute sampling windows would be obtained, the first window spanning minutes #1-10, the second minutes #2-11, and so on until the last window, spanning minutes #471-480. For each of the 471 sampling windows in this example, the four posture variables would then be calculated as estimates of their true full-shift value.

For each sampling strategy, shift and posture variable, four statistical performance variables were calculated on the basis of these estimates:

Sample bias; *B = μ_y_-M*

Sample standard deviation; *s_y_*

Lower sample dispersion limit; *D_2.5 _*= *P_2.5_-M*

Upper sample dispersion limit; *D_97.5 _*= *P_97.5_-M*

where *μ_y _*and *s_y _*are the mean and standard deviation of the sample estimates (n = 471 in the example above); *M *is the true exposure value for the full-shift; and *P_2.5 _*and *P_97.5 _*are the empirical 2.5^th ^and 97.5^th ^percentile values of the cumulative distribution of the sample estimates. Thus, *D_2.5 _*and *D_97.5 _*state the distances in either direction from the true exposure value that a single sample will exceed with a 2.5% probability, and can be viewed as measures of the combined effect of bias and imprecision. *D_2.5 _*and *D_97.5 _*give the same information as the limits of agreement in a standard Bland&Altman plot [58], however, since they are based on the empirical structure of data, they represent a more versatile approach which allows for a non-normal distribution of the difference between sample estimates and true exposure values.

For each sampling strategy and posture variable, cumulative probability distribution plots across the 73 shifts were produced for each of the four performance variables, *B, s_y_, D_2.5 _*and *D_97.5_*, as a basis for comparing statistical performance.

To further examine the nature of the sampling error, Spearman's rank correlation coefficients (with 95% confidence intervals) were determined according to standard procedures [59] between *M *and *B*, and between *M *and *s_y _*for each posture variable and sampling strategy across the 73 measured shifts.

## Results

### True posture values

**Figure 1:**
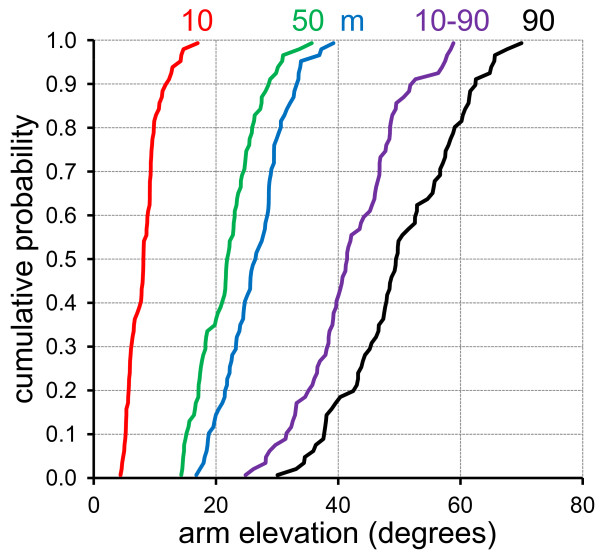
**Cumulative probability distributions across the 73 investigated full work shifts for the 10^th^, 50^th ^and 90^th ^upper arm elevation percentiles, the mean elevation (m), and the 10^th^-90^th ^percentile range, as marked by the colour codes and text above the figure**.

The true mean 10^th^, 50^th ^and 90^th ^percentile elevation angles across all 73 full-shifts in the parent data set were 8.2°, 21.9° and 50.3°, respectively, and the mean 10^th^-90^th ^percentile range was 42.0°. As expected [12], the cumulative probability distribution plots for all four posture percentile variables as well as the mean inclination angle showed a considerable dispersion across shifts (Figure 1). This exposure variability contains contributions from both between-subject and within-subject (between-shifts) sources.

### Sampling bias

**Figure 2:**
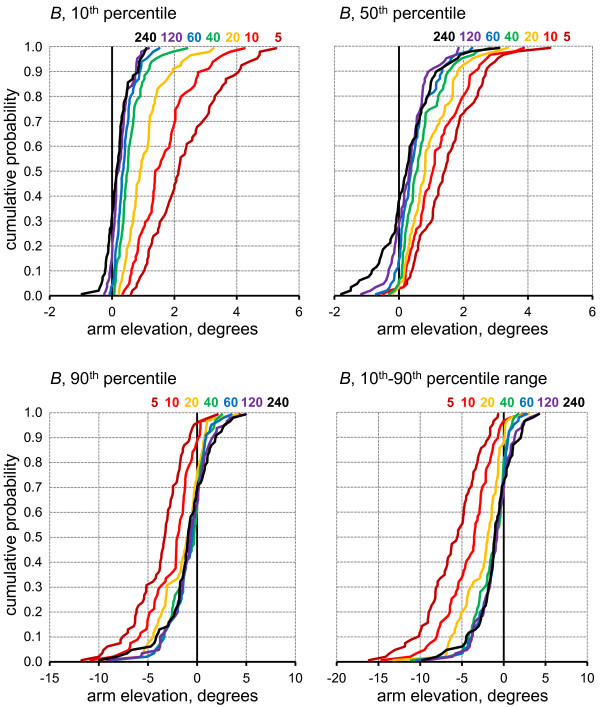
**Cumulative probability distributions across the 73 investigated work shifts for the bias, *B*, of the four assessed posture variables (10^th^, 50^th^, 90^th ^percentiles, 10^th^-90^th ^percentile range) for each of the seven sampling durations as marked by colour codes and numbers in each figure**.

Estimates of the 10^th ^and 50^th ^percentile were, in general, 'upward' biased (shifted to the right), while those for the 90^th ^percentile and the 10^th^-90^th ^percentile range were 'downward' biased (shifted to the left) for all sampling strategies; the bias was more severe with shorter sample durations (Figure 2). For a particular sampling duration, the size of the bias differed considerably among the 73 shifts (as seen in the width of the distribution) and, for long sample durations, some shifts even showed a bias in the opposite direction of the rest of the data; for example, negative values were seen for the 10^th ^and 50^th ^percentiles for sampling durations 60 min or longer, while conversely, some examples of positive bias were seen for the 90^th ^percentile and the 10^th^-90^th ^percentile range.

For the 10^th ^and 90^th ^percentiles and the 10^th^-90^th ^percentile range, bias correlated significantly with the true exposure value, *M*, in particular for short samples (Table 1). Thus, for the 10^th ^percentile variable, a larger positive bias was found for full-shifts with larger true mean exposures, while for the 90^th ^percentile and the 10^th^-90^th ^percentile range, larger true *M *values were associated with a larger negative bias. The bias of the 50^th ^percentile was positively correlated with the true exposure, *M*, value for *long *samples, but since bias was small in this case, correlations may not be particularly informative.

**Table 1:** Spearman's rank correlation coefficients [95% confidence intervals] between the true full-shift exposure value *M *and the bias *B*, and between *M *and the sample standard deviation *s_y_*, for each of the seven sampling durations (5, 10, 20, 40, 60, 120, 240 min) and each of the four posture variables (10^th^, 50^th^, 90^th ^percentiles, 10^th^-90^th ^percentile range)

Posture variable:		10^th^ percentile	50^th^ percentile	90^th^ percentile	10^th^-90^th^ percentile range
***M *vs *B***					

Sampling duration, minutes	5	**0.64** [0.48;0.76]	0.02 [-0.21;0.25]	**-0.65** [-0.76; -0.49]	**-0.66** [-0.77; -0.51]
	
	10	**0.61** [0.45;0.74]	0.03 [-0.20;0.26]	**-0.49** [-0.65; -0.29]	**-0.58** [-0.71; -0.40]
	
	20	**0.56** [0.38;0.70]	0.08 [-0.15;0.31]	**-0.27** [-0.47; -0.04]	**-0.42** [-0.59; -0.21]
	
	40	**0.53** [0.34;0.68]	**0.30** [0.07;0.49]	-0.17 [-0.38;0.06]	**-0.28** [-0.48;-0.06]
	
	60	**0.56** [0.38;0.70]	**0.28** [0.05;0.48]	-0.10 [-0.32;0.14]	-0.19 [-0.40;0.04]
	
	120	**0.57** [0.39;0.70]	**0.36** [0.15;0.55]	-0.02 [-0.25;0.21]	-0.07 [-0.29;0.17]
	
	240	**0.44** [0.23;0.61]	**0.43** [0.23;0.60]	0.09 [-0.14;0.32]	0.01 [-0.22;0.24]

***M *vs *s_y_***					

Sampling duration, minutes	5	**0.70** [0.56;0.80]	**0.65** [0.50;0.77]	**0.77** [0.65;0.85]	**0.67** [0.52;0.78]
	
	10	**0.70** [0.56;0.80]	**0.66** [0.51;0.77]	**0.73** [0.60;0.82]	**0.62** [0.46;0.75]
	
	20	**0.72** [0.58;0.81]	**0.59** [0.42;0.72]	**0.71** [0.57;0.81]	**0.60** [0.43;0.73]
	
	40	**0.68** [0.54;0.79]	**0.53** [0.34;0.68]	**0.72** [0.58;0.81]	**0.63** [0.46;0.75]
	
	60	**0.66** [0.51;0.77]	**0.52** [0.32;0.67]	**0.70** [0.57;0.80]	**0.63** [0.47;0.75]
	
	120	**0.64** [0.48;0.76]	**0.47** [0.27;0.63]	**0.67** [0.52;0.78]	**0.55** [0.37;0.70]
	
	240	**0.57** [0.39;0.71]	**0.40** [0.19;0.58]	**0.44** [0.23;0.61]	**0.36** [0.15;0.55]

In bold: correlations for which the 95% confidence interval does not contain 0.

### Sampling precision

**Figure 3:**
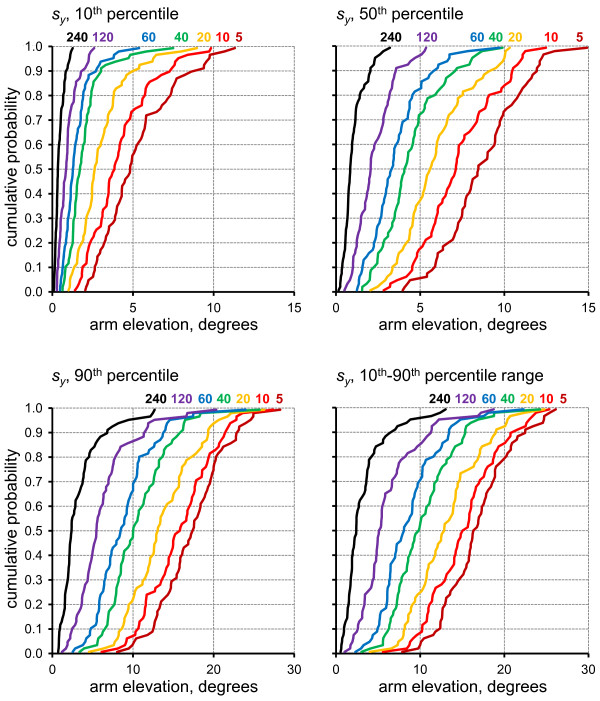
**Cumulative probability distributions across the 73 investigated work shifts for the sample standard deviation, *s_y_*, of the four assessed posture variables (10^th^, 50^th^, 90^th ^percentiles, 10^th^-90^th ^percentile range) for each of the seven sampling durations as marked by colour codes and numbers in each figure**.

As anticipated, the precision of the sample exposure estimate increased, i.e. *s_y _*decreased, with longer sampling durations for all three percentile variables (Figure 3), and for the percentile range. However, *s_y _*decreased at a slower rate than that expected for randomly distributed data, for which the standard deviation should decrease in inverse proportion to the square root of the sampling duration [35]. For all four posture variables, *s_y _*was significantly correlated with the true exposure value *M *(Table 1), and the association was stronger at short sampling durations.

### Sampling dispersion

**Figure 4:**
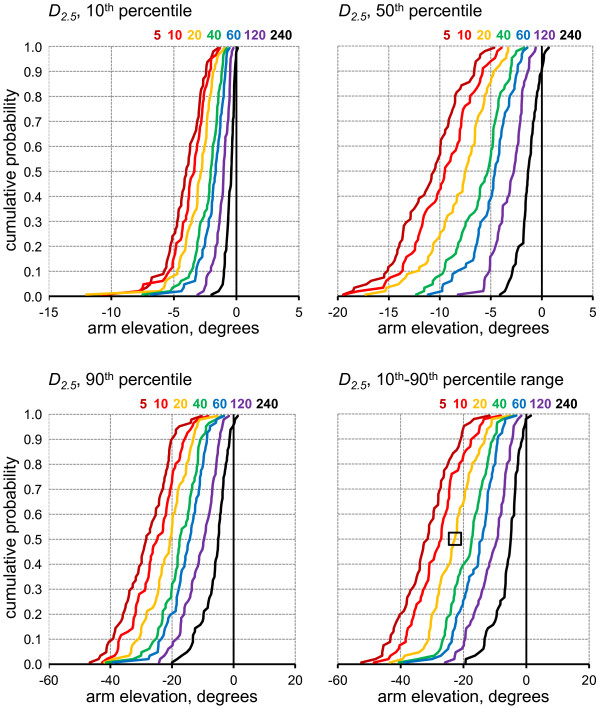
**Cumulative probability distributions across the 73 investigated work shifts for the lower dispersion limit, *D_2.5_*, of the four assessed posture variables (10^th^, 50^th^, 90^th ^percentiles, 10^th^-90^th ^percentile range) for each of the seven sampling durations as marked by colour codes and numbers in each figure**. The square marks an example described in the running text.

**Figure 5:**
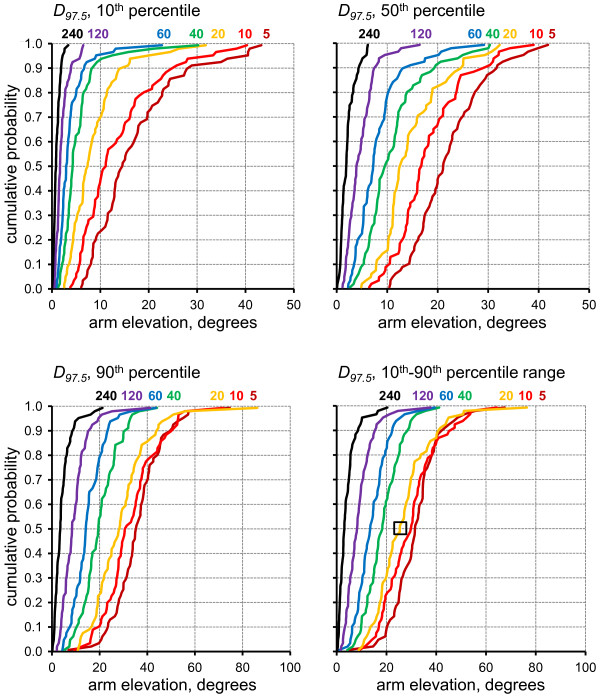
**Cumulative probability distributions across the 73 investigated work shifts for the upper dispersion limit, *D_97.5_*, of the four assessed posture variables (10^th^, 50^th^, 90^th ^percentiles, 10^th^-90^th ^percentile range) for each of the seven sampling durations as marked by colour codes and numbers in each figure**. The square marks an example described in the running text.

As expected from the precision results, smaller dispersions resulted for all postural variables with longer sampling durations, but estimates were still skewed due to the bias. This pattern is reflected in the dispersion limits *D_2.5 _*(Figure 4) and *D_97.5 _*(Figure 5) which both approach zero with increasing sample duration, while not being of equal size. Ninety-five percent of all samples within a full-shift will lie in the interval between the two dispersion limits. For example, for the 20 min sampling strategy, an estimate of the 10^th^-90^th ^percentile range could, in median, with a 5% probability be either more than 22.7° smaller (marked in Figure 4) or more than 25.4° larger (marked in Figure 5) than the true full-shift percentile range.

## Discussion

The present study showed that estimates of the 10^th ^and 90^th ^upper arm elevation percentiles and the 10^th^-90^th ^percentile range can be severely biased if they are estimated from posture samples collected over short periods of time. Since short samples were also shown to have a larger variability, i.e. be less precise, great caution should be exercised when using such sampling strategies to estimate overall exposures intended to represent extended periods of time. The findings also imply that previous studies reporting posture percentiles should be compared cautiously and only with due consideration to the fact that estimates may have been based on samples of different durations. What would appear to be a difference between groups or conditions may - in whole or part - be the result of a statistical flaw.

### Hairdressers as a representative working population

As reported by Wahlström and co-workers [12], the exposure of the present hairdressers was similar to those in a sample of Norwegian hairdressers [25], when considering that the Norwegian data were collected only when the hairdressers had customers, as compared to the current study which measured full working shifts, irrespective of task. The mean 10^th ^percentile value across the hairdressers in the present study population was similar to previously reported values from a wide range of occupational groups, including: sewing machine assembly workers [17], car disassembly workers [9], and hospital cleaners [19], while it was lower than that reported for CAD operators [60], air traffic controllers [13], floor sheet handlers [18], and dentists [21]. The 90^th ^percentile was comparable to that of cleaners [19] and dentists [21]. The 10^th^-90^th ^percentile range was somewhat smaller than that reported for car disassembly workers [9]; this variable was nor reported in any of the other cited studies. In summary, the exposures seen in the present population seem comparable to those experienced by several other occupational groups, and we therefore believe that the performance of percentile measurement strategies as reported here is valid in a wide range of occupations. The observed dependence of both the bias, *B*, and the sample standard deviation, *s_y,_*, on the true exposure level suggests that in occupational settings with more extreme percentiles than the present ones, errors associated with limited sampling would be larger than those reported here. Further, these suppositions would apply even to jobs characterized by cyclic operations, such as in industrial assembly. While it is often claimed that such cyclic jobs can be adequately monitored by collecting data from just a few work cycles, several studies suggest that the magnitude of cycle-to-cycle posture variability can, indeed, be considerable [51,61,62]. In a real occupational setting, the full-shift exposure variability will, in addition to this within-day cycle-to-cycle variability, include contributions from non-cyclic and irregular parts of the job such as production stops, breaks, occasional tasks, and meetings. Thus, the statistical performance of limited duration sampling strategies in cyclic work may not deviate as much as expected from that seen in non-routinized work [63].

### Bias, imprecision and sampling duration

Bias (Figure 2) and lack of precision, i.e. a large *s_y _*(Figure 3), was very pronounced with sampling durations of 5-10 min - durations which are not unusual among ergonomics practitioners, e.g. in occupational health practices [48]. In research studies, samples of this short a duration are rare, but the present study showed that the magnitude of both bias and imprecision was considerable even for samples of 20-120 min, as has been previously used [15-18,24]. Thus, the example marked by squared in Figures 4 and 5 illustrates that for an 'average' (median) shift, using a 20 min sampling strategy to determine the 10^th^-90^th ^percentile range would result in 5% of all estimates deviating more than 22.7° downwards or 25.4° upwards from the true full-shift value. For half of the investigated shifts, the 20 min sampling strategy performed even worse.

Short samples may occur if a 'long' job sampling period is subdivided into tasks included in the job [9,12,21,40,64]; and also in cases where a sample is subdivided in shorter sequences, for instance for illustrative purposes (see e.g. Figure 3 in [13]). In these cases, it is important to comprehend that an overall job exposure estimate obtained as a weighted average of such constituent parts [40,52] will be biased to the same extent as if sampling were conducted using windows that short.

We believe that the finding of limited sampling leading to upward biased 10^th ^percentiles and downward biased 90^th ^percentiles and 10^th^-90^th ^percentile ranges is a general result, inherent to these percentile variables, irrespective of the exposure domain. In addition to the empirical findings in the present study, we also base this contention on similar results obtained by simulating limited sampling strategies from artificial, random data sets, and on the observation by Trask et al. of biased percentiles in samples of electromyography from the lower back [56]. We also believe, on the basis of our observation that the estimation bias of the 10^th ^and 90^th ^percentiles was directed 'inwards' towards the overall central exposure value, that more extreme percentiles will be even more biased with limited sampling. Thus, we recommend great caution when assessing, comparing and interpreting extreme percentiles, for example, the 1^st ^and 99^th ^posture percentiles as reported in a recent compilation of studies from a large range of occupations [24], or 5^th^-95^th ^posture percentile ranges as used in a number of studies [13,22,38].

The present study investigated the performance of limited sampling *within *shifts because true exposures were available for comparison at this level. Since, however, posture distributions vary *between *shifts for a particular individual [12,23,40,42], true posture percentiles for single full-shifts may, themselves, be biased estimates of posture percentiles across multiple shifts, analogous to within-shift samples being biased estimates of that shift's true exposure. Thus, the magnitude of bias reported here may, in fact, underestimate the bias of using limited within-day samples as representations of the overall occupational exposure of an individual across an extended period of time, which is standard practice in epidemiologic exposure assessment. Examination of the present data set, which contained subsets of 2-4 shifts from different days belonging to the same subject, indicated that the true percentiles of individual shifts (Figure 1) were, indeed, biased when compared to the corresponding variables measured across all available shifts within the same subject; the size of the bias was, however, small compared to the sample bias within a shift. The most pronounced example of bias across shifts was seen for the 10^th ^percentile, where 16 of the 20 observed hairdressers had upwards biased shift estimates (as expected), with an average bias of 0.4°. Due to the limited number of shifts per subject, however, the extent and structure of this across-shifts bias could not be determined in detail.

### Effects of exposure bias and imprecision in epidemiologic studies

Bias and imprecision of exposure estimates have an obvious influence on the quality of studies documenting or comparing exposures in individuals or groups. Bias shifts the central result of a study and imprecision reduces its informative value and decreases power [30,44]. The combined effect of bias and imprecision can be assessed by simple metrics, such as the lower and upper dispersion limits (cf. Figures 4 and 5).

The effects of exposure bias and imprecision are less trivial in an epidemiologic investigation of the relationship(s) between exposure and outcome, for example whether the occurrence of neutral or extreme postures, or the size of posture variation, has an effect on the likelihood of developing an MSD. The general effect of within-subject imprecision is to attenuate the true exposure-outcome relationship, i.e. to modify its slope towards zero, and add uncertainty to the slope estimate [65-68]. This attenuation effect is directly dependent on the so-called variance ratio, i.e. the ratio of within- to between- subject variability; a proportionally larger within-subject variability leading to more pronounced attenuation [65,66]. For a consistently increasing exposure-outcome relationship, an upward biased exposure estimate will lead to an estimated exposure-outcome relationship that predicts the outcome at a certain exposure level to be smaller than what it actually is, but the shape of this attenuation depends on whether the bias is constant across exposure levels or proportional to the exposure, as suggested in the present data set. Thus, both bias and imprecision in 10^th ^percentile estimates lead to attenuation of an increasing exposure-outcome relationship, for instance, when investigating whether decreased occurrence of neutral postures (i.e. a larger 10^th ^percentile value) leads to a larger risk for MSD.

In the case of "extreme" postures measured through the 90^th ^percentile, an expectedly increasing relationship with an MSD outcome would be attenuated by exposure imprecision but amplified by the downward bias. Thus, bias and imprecision would have opposite effects on the estimated exposure-outcome relationship. The net trade-off must be addressed in each particular case, but in the present setting, noting the between-subjects and between-days percentile variabilities for the 90^th ^arm elevation percentiles reported by Wahlström et al. [12], the attenuating effect of imprecision seems to be more potent than the amplifying effect of bias. A similar opposite effect of bias and imprecision on the exposure-outcome relationship will appear if the exposure value is downward biased and the relationship is decreasing. This is the expected scenario if the influence of posture variation on MSD risk is addressed using the 10th-90th percentile range as the exposure metric.

While the consequences of exposure variability for exposure-outcome relationships can often be assessed and adjusted for, provided that the sources of this variability are known [66,68], adjustment for bias requires access to specific and sufficient information on the structure and properties of the bias to permit a translation of estimated exposures to the expected true values [69-71].

### Data collection strategies for posture percentile variables

In the present study, bias was correlated with true exposure; thus it might be possible to derive a useful expression to translate an estimate to the expected true value, conditional on the sampling strategy. As a more feasible correction approach, which can be used even if true values are not available for calibration, the overall median (or mean) bias calculated for a similar occupational setting using the same sampling duration (cf. Figure 2) could be used to adjust the percentile estimate.

If the residual error after bias correction does not have the desirable random and normal distribution properties, limited sampling strategies may not perform as expected from analytical theory [50,52]. For instance, exposure may be correlated within days, as when a work shift is composed of a sequence of tasks with different exposures [52], between days, as in seasonal work [72] or between subjects, as in team work [73]. In these cases, depending on the structure of the correlation, effects of increasing the sample duration can be larger or smaller than anticipated by theory [50], and consecutive sampling may perform better or worse than a more dispersed allocation of the same total sample duration [52]. For a particular setting, this behavior can be difficult to predict even if a negative effect on sampling performance may be the more common case [50].

For the present data set, visual inspection of the time-patterns of upper arm elevation angle over individual shifts clearly indicated that a non-random data structure was common: extended periods with a large upper arm elevation, when handling customers, could alternate with long periods in an almost neutral posture when no customers were present. This autocorrelation is a probable explanation to the less-than-expected decrease in *s_y _*which was observed with increased sampling duration (Figure 3). The attenuated effect of increasing the sample duration implies that the precision of a percentile estimate does not improve as much as expected from theory when longer samples are employed. In this case, sampling performance may improve if a particular sample duration is obtained with dispersed sampling rather than in a consecutive approach, as simulated in the present study [50,52]. Alternative sample distributions across time were not investigated here, and consecutive sampling has been the dominant strategy in previous studies of working postures, particularly in studies employing on-site or video observations [39,74-77]. Consecutive observation may have originally been chosen by virtue of the logistic simplicity, while the drawbacks, in terms of decreased efficiency, may not have been realized. In the case of postures assessed by inclinometry, sampling in separate time blocks within a day is not an attractive option. Efforts associated with equipment set up on the subject are substantial, and continuous monitoring with the inclinometers in place appears to be a cost-efficient choice. In this case, however, recordings should be continued for sufficiently long periods for the bias and precision to reach satisfying levels; i.e. at least 120 min when using metrics based on the 10^th ^and 90^th ^percentiles in occupational settings comparable to the one studied here.

Autocorrelation may also help explain the occasional overestimate of the 90^th ^percentile, and hence the 10^th^-90^th ^percentile range, particularly for long sampling durations (Figure 2). With randomly distributed data, overestimation of the 90^th ^percentile and the 10^th^-90^th ^percentile - and the corresponding incident of an underestimated 10^th ^percentile - would probably occur only in rare cases, if ever.

### Posture metrics - alternatives to percentiles

The 10^th ^and 90^th ^posture percentiles are intended to measure the occurrence of neutral and extreme postures, respectively, in a posture recording. A number of studies have applied alternative metrics for these purposes, based on the proportion of time spent in certain posture sectors [78]. The operational definition of neutral upper arm postures have included angles less than 20° [9,12,79], 30° [14,80] and 45° [81], and extreme or "severe" postures have been expressed as angles larger than 60° [9,12,14,23,80] or 90° [6,81]. Similar posture metrics based on time spent in specific angle sectors have also been presented for other body regions, including the neck [9,14,23,80-82], trunk [9,23,27,43,51,80,82] and wrist [9,82]. A few studies have documented the statistical performance of arm posture metrics based both on angle sectors and percentiles in the same population [12,23], suggesting posture variabilities between and within subjects to be of similar sizes for comparable variables. Thus, in this respect, angle sector metrics are a viable alternative to percentiles. Furthermore, and most important, angle sector metrics are not inherently biased. For instance, with a randomly distributed arm elevation across a shift, the mean value of all 5-minute samples for percent time with arms above 90° will be the correct value for that shift, while the corresponding 90^th ^percentile estimates will, on average, be too small (cf. Figure 2). This suggests that summary metrics based on time proportions are preferable to metrics based on percentiles, in particular if the sampling duration is short. With longer samples, the percentile bias vanishes (Figure 2), and the choice among relevant posture metrics can be based predominantly on their statistical performance in terms of precision. Thus, as a tentative rule of thumb, samples longer than 120 min allow for satisfying estimates of both 10^th ^and 90^th ^percentiles and angle sector metrics, while the percentiles should not be utilised on shorter samples.

For the 10^th^-90^th ^percentile range, alternative metrics are not as easy to identify. Other metrics assessing the "how much" aspect of variation have been proposed [35], such as, the standard deviation between mean angles in posture recording chunks throughout a shift [44], and the standard deviation of the cell values in an Exposure Variation Analysis of postures [36,37], but neither of these variables have attractive or even documented statistical properties. Thus, a viable unbiased alternative to the 10^th^-90^th ^percentile range with known statistical performance still needs to be developed.

## Conclusions

This study demonstrated the risk of encountering both substantial bias and pronounced lack of precision when estimating full-shift upper arm posture percentiles on the basis of samples of limited duration. We believe this to be an inherent property of percentiles as a class of exposure metrics, even for other measures of biomechanical exposure, including postures of other body parts and muscle activity levels measured by electromyography. This disadvantage should be noted when deciding which posture variables to use both in research studies and, in particular, in ergonomics practice, where short sampling durations may be dictated by resource limitations [48]. Alternatives to variables based on percentiles may be preferable, such as the proportion of time spent in pre-defined angle sectors [9,12,23,27,79,80]; informed decisions based on the statistical properties of such alternatives are paramount [6,12,50,51,78].

## Competing interests

The authors declare that they have no competing interests.

## Authors' contributions

SEM conceived the study concept, designed the simulation procedures, processed the results, and drafted the manuscript. JW was responsible for the full-shift parent data set collection, took part in designing the data analyses, and contributed significantly to the manuscript. MF took part in designing the data analyses, performed the numerical simulations, and contributed significantly to the manuscript. All three authors read and approved the final manuscript.

## Pre-publication history

The pre-publication history for this paper can be accessed here:

http://www.biomedcentral.com/1471-2288/12/36/prepub
